# An olive-derived elenolic acid stimulates hormone release from L-cells and exerts potent beneficial metabolic effects in obese diabetic mice

**DOI:** 10.3389/fnut.2022.1051452

**Published:** 2022-11-01

**Authors:** Yao Wang, Yajun Wu, Aiping Wang, Aihua Wang, Hana Alkhalidy, Richard Helm, Shijun Zhang, Hongguang Ma, Yan Zhang, Elizabeth Gilbert, Bin Xu, Dongmin Liu

**Affiliations:** ^1^Department of Human Nutrition, Foods, and Exercise, College of Agricultural and Life Sciences, Virginia Tech, Blacksburg, VA, United States; ^2^College of Life Sciences, Zhengzhou University, Zhengzhou, Henan, China; ^3^Department of Biochemistry, College of Agriculture and Life Sciences, Virginia Tech, Blacksburg, VA, United States; ^4^Department of Nutrition and Food Technology, Jordan University of Science and Technology, Irbid, Jordan; ^5^Department of Medicinal Chemistry, School of Pharmacy, Virginia Commonwealth University, Richmond, VA, United States; ^6^School of Animal Sciences, College of Agricultural and Life Sciences, Virginia Tech, Blacksburg, VA, United States; ^7^Department of Pharmaceutical Sciences, Biomanufacturing Research Institute and Technology Enterprise, North Carolina Central University, Durham, NC, United States; ^8^Virginia Tech Drug Discovery Center, Virginia Tech, Blacksburg, VA, United States

**Keywords:** elenolic acid, glucagon-like peptide-1, peptide YY, obesity, type 2 diabetes, mice

## Abstract

Insulin resistance and progressive decline in functional β-cell mass are two key factors for developing type 2 diabetes (T2D), which is largely driven by overweight and obesity, a significant obstacle for effective metabolic control in many patients with T2D. Thus, agents that simultaneously ameliorate obesity and act on multiple pathophysiological components could be more effective for treating T2D. Here, we report that elenolic acid (EA), a phytochemical, is such a dual-action agent. we show that EA dose-dependently stimulates GLP-1 secretion in mouse clonal L-cells and isolated mouse ileum crypts. In addition, EA induces L-cells to secrete peptide YY (PYY). EA induces a rapid increase in intracellular [Ca^2+^]_i_ and the production of inositol trisphosphate in L-cells, indicating that EA activates phospholipase C (PLC)-mediated signaling. Consistently, inhibition of (PLC) or Gα_q_ ablates EA-stimulated increase of [Ca^2+^]_i_ and GLP-1 secretion. *In vivo*, a single dose of EA acutely stimulates GLP-1 and PYY secretion in mice, accompanied with an improved glucose tolerance and insulin levels. Oral administration of EA at a dose of 50 mg/kg/day for 2 weeks normalized the fasting blood glucose and restored glucose tolerance in high-fat diet-induced obese (DIO) mice to levels that were comparable to chow-fed mice. In addition, EA suppresses appetite, reduces food intake, promotes weight loss, and reverses perturbated metabolic variables in obese mice. These results suggest that EA could be a dual-action agent as an alternative or adjuvant treatment for both T2D and obesity.

## Introduction

Type 2 diabetes (T2D) is a result of chronic insulin resistance and loss of β-cell function and mass (1–3). In both experimental animals and people, obesity is a leading pathogenic factor for developing insulin resistance and T2D, which is regarded as a significant obstacle for effective metabolic control in many patients with T2D. Constant insulin resistance will progress to T2D when β-cells become unable to secrete adequate amounts of insulin to compensate for decreased insulin sensitivity, which is largely due to loss of functional β-cell mass (1–3). Metformin has been widely used for the treatment of T2D. While it is effective in ameliorating hyperglycemia primarily by reducing hepatic glucose production and enhancing insulin sensitivity, it is unable to promote desired weight loss and stop the progressive decline in β-cell function and mass (4, 5).

It is well-established that the incretin hormone glucagon-like peptide-1 (GLP-1), which is primarily secreted from intestinal L-cells, plays a critical role in maintaining glycemic homeostasis *via* potentiating glucose-stimulated insulin secretion (GSIS) and promoting β-cell proliferation and survival (6–8). Recently, GLP-1-based drugs, including GLP-1 analogues and dipeptidyl peptidase-IV (DPP-IV) inhibitors that inhibit breakdown of GLP-1, have been developed for treating T2D (9–11), but patients given GLP-1-based drugs suffer from side effects such as nausea and vomiting (12). In addition, the weight loss efficacy of these monotherapies is often limited (13). Compared with GLP-1, peptide YY (PYY) secreted from L-cells may play a more important role in regulating satiety and food intake in both humans and rodents (14, 15). Recent studies further showed that GLP-1 and PYY synergistically inhibit appetite in mice (16) and humans (17, 18). Therefore, agents that are capable of targeting both GLP-1 and PYY secretion could be a safe and effective strategy in the prevention and treatment of T2D by promoting obesity and insulin resistance control and simultaneously improving functional β-cell mass.

A recent study showed that consumption of extra virgin olive oil (EVO) provided better glycemic control as compared with pure olive oil (POO) in human subjects (19). EVO has a nearly identical fatty acid composition but considerably higher amount of phytochemicals than POO (20), suggesting that the beneficial effect of EVO may be at least partially attributed to one or more of its phytochemical components. Interestingly, EVO intake resulted in higher serum GLP-1 and insulin levels but lower postprandial glycemia relative to POO in patients with morbid obesity (19). However, the specific compound(s) that are responsible for the observed metabolic beneficial actions of EVO are unclear. Oleuropein and its derivatives such as hydroxytyrosol and elenolic acid (EA) are major phytochemicals in olives (21). EA is present in mature olives and EVO, and is derived from decomposition of oleuropein during fruit maturation (22). Oleuropein and its aglycone oleacein have moderate glucose-lowering effects and ameliorate high-fat-diet (HFD)-induced metabolic alterations in rodent models, but it has not been investigated either *in vitro* or *in vivo* whether EA exerts beneficial actions related to healthy or disease states such as diabetes. In this study, we generated EA by hydrolyzing oleuropein and investigated the effects of EA on GLP-1 and PYY secretion in L-cells and further explored the underlying mechanism of EA’s action. In addition, we explored the anti-diabetic effects of EA in diet-induced obese (DIO), glucose intolerant mice.

## Materials and methods

### Chemicals and reagents

Oleuropein (purity ≥ 80%, HPLC) for EA synthesis was from Shaanxi Huike Botanical Development Co. (Xi’an, China); metformin was from Cayman Chemicals (Ann Arbor, MI, USA); U73122 was from Tocris Bioscience (Pittsburgh, PA); YM 254,890 was from Focus Biomolecules (Plymouth Meeting, PA, USA); DMEM media, fetal bovine serum (FBS), and other cell culture supplements were from Hyclone (GE Healthcare Bio-Sciences, Pittsburgh, PA, USA); fluro-4AM was from ThermoFisher Scientific (Waltham, MA); antibodies for western blotting were from Santa Cruz Biotechnology (Dallas, TX, USA); assay kits for measuring active GLP-1 [GLP-1 (7–36) amide and GLP-1 (7–37)], triglyceride, human PYY as well as dipeptidyl peptidase IV (DPP4) inhibitor assay kit were from Cayman Chemical (Ann Arbor, MI); oleacein, hydroxytyrosol, oleocanthal, tyrosol, and oleuropein (≥ 95% pure) for GLP-1 secretion assays, RPMI1640 medium, and all other chemicals were purchased from Millipore Sigma (Burlington, MA, USA); IP_3_ ELISA kit was from Amsbio (Cambridge, MA, USA); mouse insulin and leptin ELISA kits and mouse total GLP-1 ELISA kits were from Crystal Chem (Elk Grove Village, IL); glucose meter and strips were from AgaMatrix (Salem, NH); and free fatty acid (FFA) assay kits were from BioAssay Systems (Hayward, CA, USA).

### Generation of elenolic acid

Oleuropein was dissolved in tetrahydrofuran (THF), followed by the addition of 1 N aqueous H_2_SO_4_ and stirring for 48 h at room temperature. After removing THF under vacuum, the solution was neutralized to pH 7.5 with 1 N NaOH solution. The solution was washed with ethyl acetate (EtOAc) three times. The neutral solution was acidified with 1 N HCl and extracted with EtOAc. The organic layer was collected and dried with anhydrous sodium sulfate overnight. After evaporating the solvent, the residue was purified by flash chromatographic procedures, using dichloromethane-methanol (contain 1% acetic acid) as mobile phase to obtain EA. The final product identity and purity was confirmed by liquid chromatography-mass spectrometry (LC-MS) (SHIMADZU Scientific Instruments, Inc., Columbia, MD, USA), ^1^H-NMR, and ^13^C-NMR analyses.

### Glucagon-like peptide-1 secretion measurement

GLUTag L-cells (provided by Dr. Daniel J. Drucker, Lunenfeld Tanenbaum Research Institute, Toronto, ON, Canada) were cultured as previous described (23). For GLP-1 secretion assays, cells were incubated with Krebs-Ringer Modified (KRB) buffer containing 0.2% bovine serum albumin (BSA) for 30 min and then treated with EA (0.1, 1, or 10 μM), 10 μM oleacein, hydroxytyrosol, oleocanthal, tyrosol, oleuropein, or 1 μM forskolin and 1 μM 3-isobutyl-1-methylxanthine (IBMX) for 1 h. For some experiments, cells were pretreated with various inhibitors for 30 min prior to the addition of 10 μM EA for 1 h. Supernatants were collected for measuring GLP-1 [7–36 (NH2) and GLP-1 (7–37)] concentrations using an assay kit, which was then normalized to the protein content in the same sample. The ileum crypts were isolated from mice as previously described (24, 25). The crypts were maintained for 48 h, and then treated with EA or forskolin (10 μM) plus IBMX for 1 h to measure GLP-1 secretion.

### Peptide YY secretion assay

NCI-H716 cells (ATCC, CCL-251) were maintained in RPMI1640 medium supplemented with 10% fetal bovine serum (FBS), 2 mM L-Glutamine, and 1% penicillin streptomycin in a humidified incubator at 37°C and 5% CO_2_. For PYY secretion, 2 × 10^5^ NCI-H716 cells were seeded into a 12-well plate that was precoated with Matrigel. Before the treatment, cells were incubated with KRB buffer containing 0.2% BSA for 30 min and then treated with EA or 2 mM butyrate for 1 h. Supernatants were collected for measuring PYY (in the form of 3–36) concentration using an assay kit, which was then normalized to the protein content in the same sample.

### Intracellular calcium measurement

To identify whether EA induces intracellular calcium influx, GLUTag cells were loaded with 2 μM Fluo-4AM in Ca^2+^-free KRB and incubated at 37°C for 1 h. The cells were then washed and resuspended in Ca^2+^-free KRB buffer at 2 × 10^6^ cell/ml and transferred to opaque 96-well microplates. Basal fluorescence signals of Fluro-4AM-loaded cells were measured at 495 nm excitation and 518 nm emission using a spectrofluorophotometer (FLUOstar OPATIMA, Cary, NC, USA). After 10 sec, EA or inhibitor was injected into culture plates, and fluorescence was continuously recorded for 240 s.

### Inositol trisphosphate assay

GLUTag cells (10^5^ cells/well) were treated with vehicle or 10 μM EA for 30 sec, followed by addition of 10% perchloric acid to terminate the reaction. IP_3_ concentrations in the cell lysates were measured using an ELISA kit.

### Trypsin protection assay

To investigate whether EA binds with Gα subunits, the isolation of plasma membranes and subsequent trypsin protection assays were performed as described (26) with modifications. The inactive Gα can be readily cleaved by trypsin, whereas the active Gα due to the agonist-stimulated binding of GTP protects it from trypsinolysis (26). In brief, GLUTag cells were lysed in 10 mM Tris–HCl (pH 7.4) buffer containing 5 mM EDTA, 10 μg/ml benzamidine, 10 μg/ml soybean trypsin inhibitor (type II-S), and 5 μg/ml leupeptin, and plasma membranes were isolated. Cell membranes (30 μg) were then incubated in 25 mM HEPES (pH 7.5) buffer containing 1 mM EDTA, 20 mM β-mercaptoethanol, 25 mM MgCl_2_, 100 mM NaCl, 10 μM GDP, and 50 μM GTPγS in the presence or absence of EA or vehicle at 37°C for 15 min. Afterward, the cell membranes were digested with 100 μg/ml of N-tosyl-L-phenylamine chloromethyl ketone (TPCK)-trypsin (1:25 ratio of trypsin to total protein) at room temperature for 15 min, and the reaction was terminated by the addition of gel loading buffer and heating at 95°C. The samples were then analyzed by immunoblotting with antibodies against Gα_*s*_, Gα_i_, or Gα_q_.

### Animals

Male C57/BL6J mice (male, 8 weeks old) were purchased from the Jackson Laboratory (Bar Harbor, ME, USA). All mice were kept under constant temperature (23°C) and Light cycle (12 h Light/12 h dark) with *ad libitum* access to water and either a standard chow diet (SD, 11% kcal from fat) or high-fat-diet (HFD, 58% kcal from fat) (Research Diets, Inc., New Brunswick, NJ, USA). All protocols for the following animal experiments were approved by the Institutional Animal Care and Use Committee at Virginia Tech.

### Acute effects of elenolic acid on glucagon-like peptide-1 and peptide YY secretion and glucose intolerance in mice

Next, we used DIO mice to assess whether acute administration of EA improves glucose tolerance, thereby exerting potential anti-diabetic potential consistent with its stimulatory action in GLP-1 and PYY secretion. In that regard, C57B6 mice (male, 8 weeks old) were fed a control, standard chow diet (SD; Research Diets, NJ, containing 11, 73, and 16% kcal from fat, carbohydrate and protein, respectively) or a HFD (containing 58% kcal from fat) for 12 weeks (wks) to induce obesity, insulin resistance, and glucose intolerance (27). To perform intraperitoneal glucose tolerance test (IPGTT), mice (*n* = 8/group) were fasted for 14 h followed by measuring basal blood glucose levels using samples drawn from tail vein. Mice were then given 50 mg/kg EA or same amount of vehicle *via* oral gavage. EA was dissolved in 0.1 M NaHCO_3_ and then suspended in 2% methylcellulose before administration. At 30 min after EA administration, blood samples were drawn for glucose measurements, and then an IPGTT (1.5 g glucose/kg) was performed. The area under the curve (AUC) from these tests was calculated as previously described (28). For assessing the effects of EA on GLP-1 and PYY levels, obese mice were fasted for 6 h and blood samples from tail vein were collected into pre-chilled tubes containing 50 μM diprotin A and 5 mM EDTA for baseline analyses. Mice were then administered 50 mg/kg EA or vehicle (*n* = 8/group) and blood was drawn at 15 min post-injection for measuring total GLP-1 (tGLP-1, C) and PYY (D) levels using ELISA kits. To examine the effect of EA on glucose stimulated insulin secretion (GSIS), mice were fasted for 6 h and then administered 1.5 g/kg glucose *via* IP injection. Tail vein blood samples were obtained before and 30 min after glucose administration, and plasma insulin levels were measured with an ELISA kit.

### Metabolic effects of elenolic acid on obese mice

C57BL6 mice (8 weeks old, male) were fed either a SD or a HFD (*n* = 8 mice/group) for 10 weeks to induce obesity. Obese mice were divided into two groups with similar body weight (BW) and blood glucose and then given either EA (50 mg/kg once daily) or vehicle *via* oral gavage for 2 weeks. Age-matched mice fed a SD were used as healthy controls and were administered the vehicle orally for 2 weeks. Body weight, non-fasting blood glucose (NFBG), and fasting blood glucose (FBG) were recorded weekly. For measuring food intake, pre-weighed food pellets were provided and replaced twice a week, and cumulative food intake was calculated. IPGTT was performed at the end of this experiment. Body composition of mice was evaluated by NMR Analyzer for small animals (Bruker, Billerica, MA, USA) at the beginning and end of the study. Following these procedures, mice were fasted overnight, euthanized, an blood was collected immediately *via* cardiopuncture. Plasma insulin, leptin, FFA and triglycerides were measured using assay kits.

### Statistical analysis

Data were analyzed by unpaired student’s *t*-test or one-way ANOVA using SigmaPlot software. Significant differences between multiple groups were subjected to Tukey’s test. A *p* < 0.05 was considered statistically significant. Values are presented as mean ± standard error (SE) or SE of mean (SEM), where appropriate.

## Results

### Synthesis and validation of elenolic acid

Elenolic acid (EA) was synthesized by hydrolysis of oleuropein followed by multiple steps of extraction and purification procedures. The purity of generated EA was > 95% as determined by LC-MS analysis, which showed that the predominant EA peak has a retention time of 5.50 min [M-H]^–^ of 241.07 (Supplementary Figure 1A). The isolated EA is an aldehyde form as confirmed with high-resolution mass spectrometry fragmentation (Supplementary Figure 1B), and its chemical structure and calculated mass are shown in the inset. The aldehyde form of EA was further determined by ^13^C and ^1^H nuclear magnetic resonance (NMR) spectroscopy (Supplementary Figures 1C,D). The chemical shift of aldehyde EA is 13C (> 200 ppm) and 1H (9.7 ppm) from ^13^C-NMR to ^1^H-NMR analysis, respectively.

### Elenolic acid is an intestinal L-cell functional agonist with anti-diabetic potential

**FIGURE 1:**
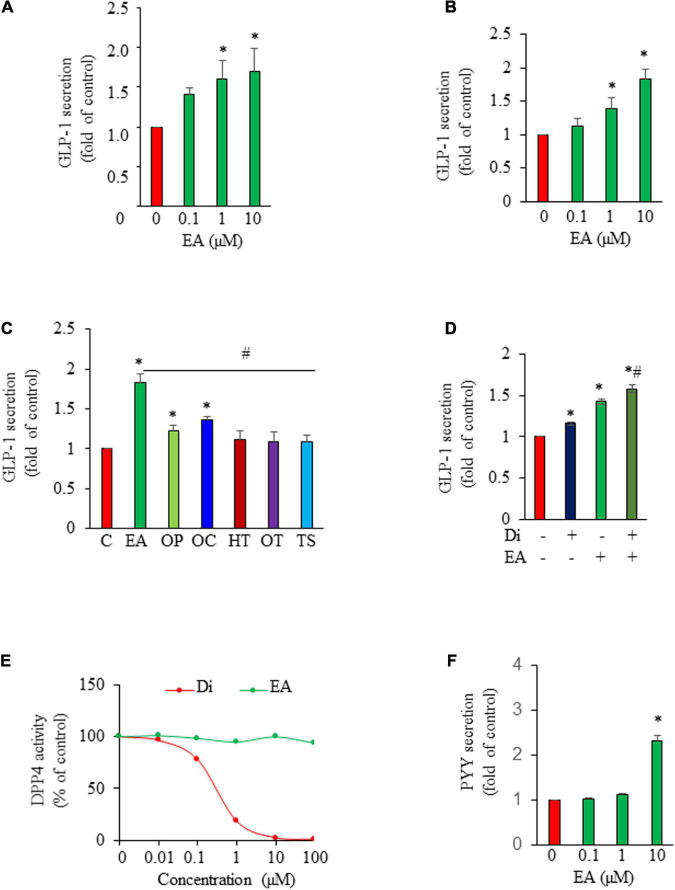
Elenolic acid (EA) induces both glucagon-like peptide-1 (GLP-1) and peptide YY (PYY) secretion from L-cells. GLUTag L-cells **(A)** or mouse ileum crypts **(B)** were incubated with various concentrations of EA or vehicle for 1 h. GLP-1 secreted into medium was measured by ELISA, and the GLP-1 secretion was normalized to protein content of each treatment and converted to the percentage of the control. **(C)** GLP-1 secretion from cells treated with 10 μM EA, oleuropein (OP), oleacein (OC), hydroxytyrosol (HT), oleocanthal (OT), or tyrosol (TS) for 1 h. **(D)** GLUTag cells were pre-incubated with or without DPP4 inhibitor vildagliptin (Di; 50 μM) for 30 min and then treated with vehicle or 10 μM EA for another 1 h. Supernatants were collected for GLP-1 secretion measurement. **(E)** The DPP4 activity in the presence of EA or DI was analyzed using a fluorescence reader at an excitation wavelength of 360 and emission wavelength of 465. **(F)** NCI-H716 L-cells were incubated with EA or vehicle for 1 h. PYY released into supernatants were measured using an assay kit. Data are mean ± SEM (*n* = 3–4). *p < 0.05 vs. control; #*p* < 0.05 vs. EA.

We show that EA is an incretin secretagogue, with 1–10 μM concentration inducing significant GLP-1 release from GLUTag L-cells after 1 h of exposure (Figure 1A). Similarly, EA treatment significantly increased GLP-1 secretion from isolated mouse ileum crypts (Figure 1B), suggesting that the EA effect on L-cells is physiologically relevant. Next, we compared the effect of EA on GLP-1 secretion with that of other secoiridoids, including oleuropein (OP), oleacein (OC), hydroxytyrosol (HT), as well as oleocanthal (OT) and tyrosol (TS). We found that EA is much more potent in stimulating GLP-1 secretion than OP and OC, whereas OT, HT, and TS are inactive (Figure 1C), indicating that EA is unique in inducing GLP-1 secretion. To determine whether EA induces GLP-1 secretion through inhibition of DPP-IV, we pre-incubated the cells with 50 μM vildagliptin, a DPP-IV inhibitor. As shown in Figure 1D, vildagliptin alone increased GLP-1 level by 16% (*p* < 0.05), which was only about 30% of that achieved by exposure to EA. In the presence of vildagliptin, EA-stimulated GLP-1 release was further increased, which should be due to the inhibitory effect of vildagliptin on GLP-1 degradation. We further confirmed that vildagliptin was very potent in inhibiting DPP-IV with doses > 10 μM completely ablating its enzymatic activity while EA at the same doses was inactive (Figure 1E). These results demonstrate that EA action on GLP-1 secretion from L-cells is mediated *via* a DPP-IV-independent mechanism. Last, to examine whether EA also directly induces PYY secretion from L-cells, we cultured NCI-H716 cells in the presence or absence of EA. Consistent with its effect *in vivo*, EA stimulated PYY secretion from L-cells, with 10 μM increasing PYY secretion by over 100% (Figure 1F).

### Elenolic acid stimulates glucagon-like peptide-1 secretion through a Gα_q_-PLC-IP_3_-Ca^2+^-dependent mechanism

**FIGURE 2:**
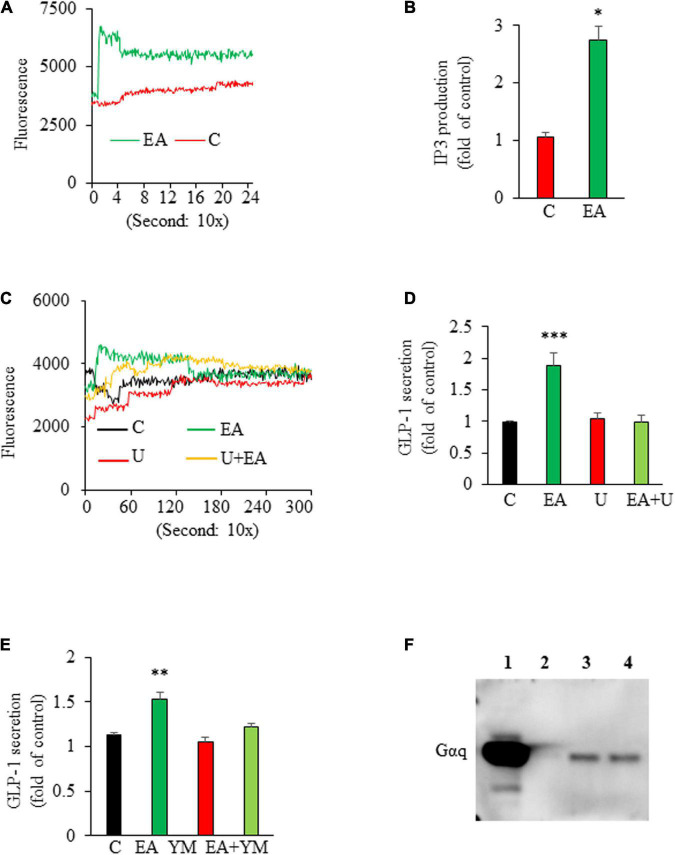
Elenolic acid (EA)-induced glucagon-like peptide-1 (GLP-1) secretion is mediated *via* a Gαq-PLC-IP3-Ca^2+^-dependent mechanism. **(A)** GLUTag L-cells were pretreated with Fluo-4AM and treated with vehicle or 10 μM EA. The [Ca^2+^]_i_ response was measured using a fluorescence plate reader. A representative graph from 4 experiments is shown. **(B)** L-cells were treated with 10 μM EA or vehicle for 20 s. The intracellular IP_3_ contents were measured using an ELISA kit. **(C)** GLUTag cells were labeled with Fluo-4 AM in the presence or absence of 10 μM U73122 (U) for 30 min, followed by an injection of 10 μM EA or vehicle. The [Ca^2+^]_i_ response was measured as stated above. **(D)** GLUTag cells were pretreated with 10 μM U73122 (U) for 30 min, followed by addition of 10 μM EA or vehicle for another 1 h. Supernatants were collected for GLP-1 measurement. **(E)** L-cells were preincubated with 10 μM Gα_q_ inhibitor, YM 254,890 (YM) for 30 min followed by incubation with 10 μM EA or vehicle for 1 h. Supernatants were collected for measuring GLP-1 secretion. **(F)** Cell membranes isolated from L-cells were treated with vehicle or EA for 15 min. Western blotting was performed with Gα_q_ antibody. L, ladder; Lane 1: assay control (without trypsin); Lane 2: vehicle; Lane 3: 1 μM EA; Lane 4: 10 μM EA. Data are means ± SEM; *n* = 5–6. *P < 0.05; **P < 0.01; ***P < 0.001 vs. control.

To explore the signaling mechanism that mediated EA-stimulated GLP-1 secretion, we first examined whether EA increases intracellular [Ca^2+^]_i_, which is critical for triggering GLP-1 secretion (29). We found that L-cells exposed to EA displayed a rapid increase in intracellular [Ca^2+^]_I_ (Figure 2A). We then examined the effect of EA on the activity of phospholipase C (PLC), which hydrolyzes phosphatidylinositol 4,5-bisphosphate (PIP_2_) to the Ca^2+^-mobilizing second messenger inositol triphosphate (IP_3_), thereby elevating cytosolic [Ca^2+^] (30). The result showed that EA treatment elicited a rapid IP_3_ production in L-cells, indicating that EA stimulated PLC activity (Figure 2B). Further, incubation of L-cells with U73122, a specific antagonist of PLC, completely ablated EA-elicited intracellular [Ca^2+^]_i_ increase (Figure 2C) and GLP-1 secretion (Figure 2D), suggesting that EA induced-GLP-1 secretion was mediated *via* the PLC-dependent pathway. Next, we determined whether EA induces GLP-1 secretion *via* Gα_q_, as the secretion of GLP-1 could be regulated *via* G-protein-coupled receptors, which signal primarily through Gα_q_, leading to activation of PLC/Ca^2+^ signaling (31–35). We found that blockage of Gα_q_ with the Gα_q_ specific inhibitor, YM 254890, diminished EA-induced GLP-1 secretion (Figure 2E). We further examined whether EA activates Gα_q_ by performing a trypsin sensitivity assay (26), Cell membranes incubated with EA exhibited a clear and strong Gα_q_ band, which was completely absent in vehicle-treated plasma membranes (Figure 2F). Collectively, these results provide evidence that EA-stimulated hormone secretion from L-cells is mediated by activating Gα_q_/PLC/Ca^2+^ signaling.

### Acute administration of elenolic acid improved glycemic control and increased blood glucagon-like peptide-1, peptide YY, and insulin levels

**FIGURE 3:**
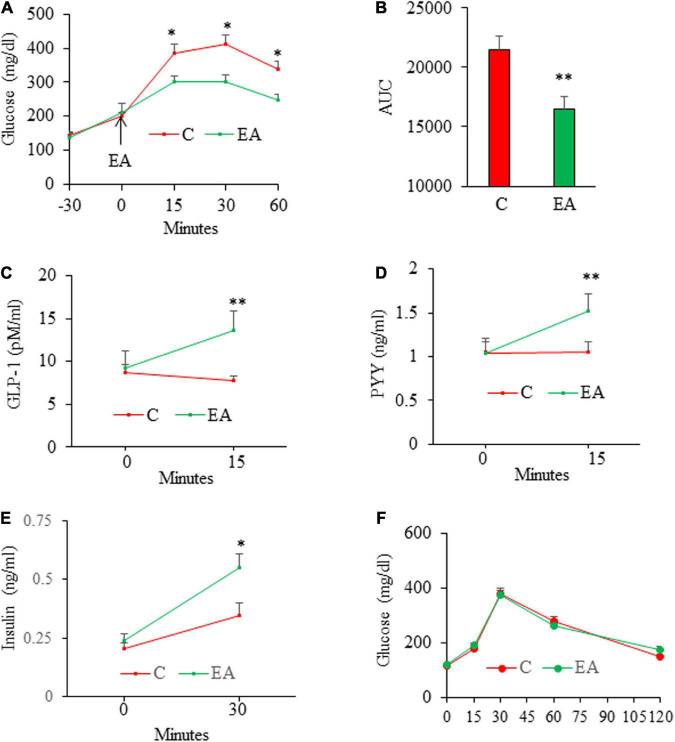
Acute effects of elenolic acid (EA) on glucose tolerance, glucagon-like peptide-1 (GLP-1), peptide YY (PYY), and insulin secretion in mice. C57BL6 male mice (8 weeks old) on high-fat-diet (HFD) for 12 weeks were fasted for 14 h and then were given EA (50 mg/kg, oral gavage) or vehicle, followed by performing intraperitoneal glucose (1.5 g/kg) tolerance test (IGTT) **(A)**, and the area under the curve (AUC) from GTT was calculated **(B)**. For assessing the effects of EA on GLP-1 and PYY levels, obese mice were fasted for 6 h and blood was drawn for baseline analyses. Mice were then given EA (50 mg/kg) or vehicle. Blood total GLP-1 [tGLP-1, **(C)**] and PYY **(D)** levels were measured at 15 min after gavage. **(E)** Plasma insulin levels in 6-h fasted mice before (0) and 30 min following sequential administration of EA (50 mg/kg, gavage) and by glucose (1.5 g/kg, ip. Injection). The results of IPGTT from lean **(F)** mice following administration of EA (50 mg/kg) *via* gavage. Data are mean ± SE. *p< 0.05, **p< 0.01 (*n* = 4–8 mice/group).

We next assessed the acute effect of EA on glucose excursion in HFD-induced obese, glucose intolerant mice. The results from IPGTT showed that administration of EA significantly reduced blood glucose levels at 15, 30, and 60 min post-injection of glucose as compared with the control mice (Figures 3A,B), which is consistent with the established role of GLP-1 in glucose homeostasis. Next, we determined the effect of oral administration of EA on GLP-1, PYY, and insulin secretion in obese mice under glucose challenge. Acute administration of EA increased blood GLP-1 (Figure 3C) and PYY (Figure 3D) levels by about 50% (p < 0.05), demonstrating that EA-evoked GLP-1 and PYY (3–36) secretion from L-cells *ex vitro* is recapitulated *in vivo*. Acute administration of EA also increased plasma insulin levels in obese mice under glucose challenge (Figure 3E), suggesting that it promoted glucose-stimulated insulin secretion (GSIS), which is consistent with such a role of GLP-1 in pancreatic beta-cells. Importantly, EA did not alter blood glucose levels in SD-fed lean mice (Figure 3F), indicating that it has no hypoglycemic effect in metabolically healthy mice.

### Elenolic acid reverses hyperglycemia in diet-induced obese mice

**FIGURE 4:**
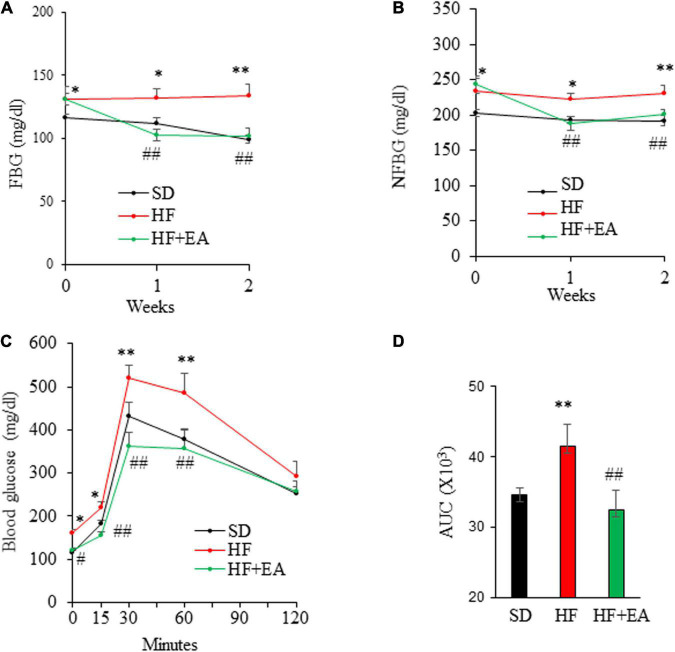
Elenolic acid (EA) is a potent anti-diabetic agent in high-fat diet (HF)-induced obese mice. C57BL6 mice (8 weeks old, male) were fed either a SD or a HF for 10 weeks, followed by treatment with EA (50 mg/kg/d) or vehicle *via* oral gavage for 2 weeks. Fasting blood glucose (FBG) **(A)** and non-fasting blood glucose (NFBG) **(B)** were measured once per week. Blood glucose levels during IPGTT (1.5 g glucose/kg) **(C)** and the area under the curve (AUC) was calculated **(D)**. Data are Mean ± SE (*n* = 8). *p < 0.05, and **p < 0.01 vs. SD-fed mice; ^#^*p* < 0.05; and ^##^*p* < 0.01 vs. high-fat-diet (HF) alone-fed mice.

We then conducted an animal study to evaluate whether EA has potential as a therapeutic for the treatment of obesity and T2D. In that regard, C57B6 mice were fed a SD or a HFD for 10 wks to induce obesity, insulin resistance, and glucose intolerance (27), followed by oral administration of EA (50 mg/kg) once per day for 2 weeks. Remarkably, treatment with EA completely reversed hyperglycemia (Figures 4A,B). Consistently, EA also fully corrected glucose intolerance (Figures 4C,D) in obese mice.

### Elenolic acid promotes body weight loss and reverses obesity-associated perturbation of metabolic variables in diet-induced obese mice

**FIGURE 5:**
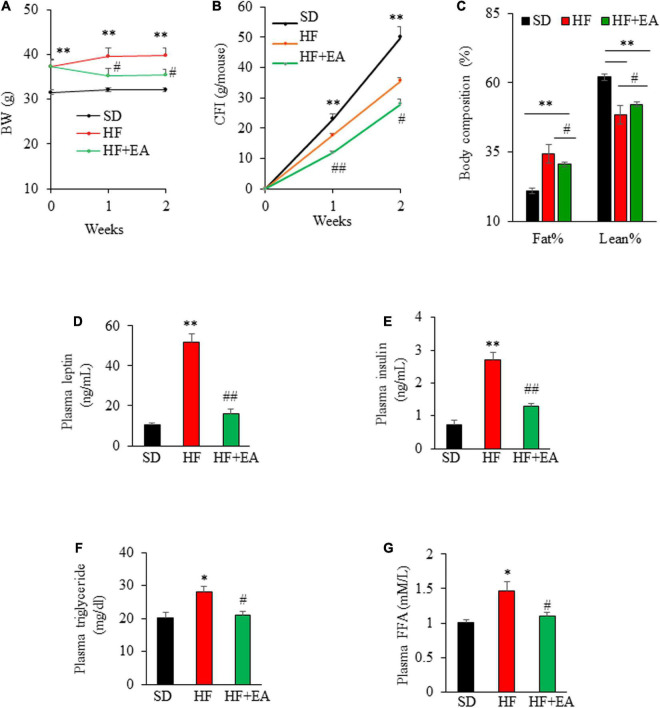
Elenolic acid (EA) promotes weight loss and reverses obesity-associated biochemical alterations. Weekly body weight **(A)** and cumulative food intake **(B)** of obese mice on the vehicle or EA treatment for 2 weeks as described in Figure 5. Body composition was measured at the end of the experiment **(C)**. Effects of EA treatment for 2 weeks on fasting plasma leptin **(D)**, insulin **(E)**, triglyceride **(F)**, and free fatty acid [**(G)**, FFA] levels in obese mice as measured using ELISA kits. Data are Mean ± SE (*n* = 8). *p < 0.05, and **P < 0.01 vs. SD-fed mice; #*P* < 0.05; and ##*p* < 0.01 vs. high-fat-diet (HF) alone-fed mice.

Oral administration of EA promoted weight loss in DIO mice during 2 wks of treatment, which reduced BW from 37.3 ± 1.4 g to 35.4 ± 1.42 g, corresponding to a decrease of 5.0 ± 1.4% (*P* < 0.05), whereas the BW of vehicle-treated DIO mice increased by 6.4% over their initial BW, 37.3 ± 1.4 (Figure 5A). In addition, EA reduced food intake, with cumulative food intake at the end of 2 weeks reduced to 28.0 ± 1.5 g/mouse in EA-treated mice from 35.6 ± 1.1 g/mouse in control group, corresponding to a 21.4% reduction (Figure 5B). The result of body composition analysis showed that EA treatment significantly reduced fat percentage, whereas the lean mass was increased by 8% relative to vehicle-treated mice (Figure 5C), suggesting that EA is likely a non-toxic, novel anti-obesity agent. As expected, blood leptin and insulin levels in obese mice were greater than in SD mice (*p* < 0.01), demonstrating that obese mice developed insulin resistant (36). Strikingly, treatment with EA for only 2 weeks reduced the obesity-elevated leptin and insulin levels by 69.3% (Figure 5D) and 58.5% (Figure 5E), respectively, in obese mice, which is likely due to improved obesity and insulin resistance consistent with previous findings in this mouse model (37, 38). EA almost completely reversed the elevated plasma triglyceride concentrations in obese mice to those in SD mice (Figure 5F; *P* < 0.05). Similarly, plasma FFA concentrations in EA-treated mice were lower relative to control obese mice (Figure 5G; *P* < 0.05).

## Discussion

While many naturally occurring compounds have been explored for their potential anti-obesity and anti-diabetic properties, the ones with both strong hypoglycemic and anti-obesity activities are rarely discovered. In the present study, we found for the first time that an olive-derived small molecule EA exerts potent glucose-lowering activity while also suppressing food intake and BW gain in DIO mice. On a cellular level, EA directly stimulates intestinal L-cells to release GLP-1 and PYY. Therefore, EA could be a potent dual-action, anti-diabetic and anti-obesity compound.

Secretion of GLP-1 and PYY from L-cells in the intestine is increased in response to ingested macronutrients, primarily fatty acids (31, 32, 39), although glucose (40, 41), some amino acids, and dietary fibers (42) may also induce hormone release. In addition, a variety of neurotransmitters and neuropeptides released by the enteric nervous system and enteroendocrine cell types, such as acetylcholine (43) and gastrin-releasing peptide (44), have been implicated in the regulation of GLP-1 secretion. However, no therapeutic strategy based on these stimuli has been successfully developed for treating T2D. While GLP-1-based drugs are effective for treating T2D, there are safe concerns that these drugs may induce gastrointestinal adverse events (45) and acute gallbladder disease (46). In addition, GLP-1 analogs are not potent in BW control and do not mimic endogenous GLP-1 secretory pattern in response to a meal intake (47). In the present study, we sought to identify agents to induce both endogenous GLP-1 and PYY secretion, thereby providing a safe and alternative strategy for obesity and T2D management. We provide strong evidence from studies of cells, intestinal tissues, and mice that EA is a novel GLP-1 and PYY secretagogue. We then found that acute administration of EA greatly improved glucose disposal in response to glucose challenge. Interestingly, acute administration of EA had no effect on glucose excursion of lean mice during glucose tolerance test, which is in line with some previous findings (48, 49) but not others (50, 51) from examining GLP-1 analogs. The lean mice used in the present study are young adult that are metabolically healthy with presumably stronger counterregulatory response to hyperglycemia as compared with obese mice. It is possible that EA-induced GLP-1 secretion and subsequent increase in circulating insulin didn’t reached levels high enough to significantly reduces blood glucose in lean mice under glucose loading. Indeed, it was found that at least 10 pM/L of GLP-1 analogs in the blood is required for achieving significant glucose lowering effect in lean mice (52). Nevertheless, using DIO mouse models, we show that EA treatment for 2 weeks already nearly reversed metabolic abnormalities caused by long-term feeding with HFD in obese mice consistent with expected metabolic effects of GLP-1 and PYY, thus providing evidence that this agent may have substantial therapeutic potential for T2D. However, it should be noted that this is only a short-term treatment (due to limited quantity of EA generated in our lab at that time) using a DIO mice that are insulin resistant and glucose intolerant, but are not overt diabetic. Therefore, the persistence and efficacy of EA in in treating obesity and diabetes need to be confirmed through a long-term study using overt obese diabetic rodent models.

The EA dose (50 mg/kg/day *via* gavage) used in this study is likely pharmacological, as it may be impossible to get this amount of EA by dietary intake of olive products, given that the content of EA in mature olives is 7–12 mg/g dry weight, and 5–22 mg/kg in extra olive oil (53, 54). We chose this dose of EA, which is equivalent to dietary intake of about 243 mg/day for a 60 kg human as calculated according to an allometric scaling method for converting doses between species (55), because it is within the dosage range of previously tested various bioactive compounds in mouse studies as well as in clinical trials. However, the optimal dose and its safety profile are still unknown, which can be identified by determining the dose-effect relationship in combination with evaluating its pharmacokinetics.

One of the most interesting observations from this study is that EA elicited robust reductions in food intake. While several naturally occurring compounds have been reported to change food palatability due to their bitter taste (56), which may modulate the reward system in the brain, thereby affecting food intake (57), we believe that EA suppression of food intake should not be secondary to the induction of taste aversion, given that EA was administered *via* oral gavage, resulting in the direct passage of EA to the stomach without inducing taste in the mouth. Rather, EA regulation of food intake is at least partially executed through stimulated release of the metabolic hormones from the gut. Food intake is controlled primarily by a variety of orexigenic and anorexigenic signals that are integrated in the hypothalamus. It was recently found that the GLP-1 receptor is expressed in several brain regions such as the paraventricular nucleus (PVN) and the arcuate nucleus (ARC) of the hypothalamus, and that GLP-1 targets control circuits in these regions to induce satiety and reduce food intake (58). PYY is an anorectic hormone that regulates gut mobility and directly suppresses appetite in the brain *via* an Y2 receptor-mediated pathway (16). It was found that mice deficient in PYY are hyperphagic and obese while PYY replacement restored their lean phenotype (15). In addition, PYY may also promote β-cell function and survival (59), thereby contributing to maintaining glucose homeostasis. In addition to stimulating GLP-1 secretion *in vitro* and *in vivo*, we found that EA also greatly increased circulating PYY in mice and directly stimulated PYY release from cultured L-cells, which should be a major contributing factor for the hypophagic and anti-obesity effects of EA treatment. Previous studies show that GLP-1 and PYY synergistically inhibit appetite in mice (16) and humans (17, 18), suggesting that simultaneous activation of these gut hormones might work additively to provide better metabolic outcomes than those achieved by GLP-1 or its receptor agonist alone. Thus, it is intriguing to test in the future whether EA could be potentially a more effective anti-diabetic and anti-obesity agent than some of the currently prescribed monotherapeutic drugs such as DPP4 inhibitors, metformin, and GLP-1 analogs. In addition, as several other intestinal hormones secreted in response to meal ingestion, such as glucose-dependent insulinotropic polypeptide (60) and cholecystokinin (61), also play a role in promoting satiety, studies of transgenic mouse models combined with analyses of hypothalamic pathway controlling food intake are needed to determine whether EA regulation of food intake and body weight is exclusively mediated *via* GLP-1 and PYY-mediated mechanisms.

In the present study, we performed a series of experiments *in vitro* with the results indicating that EA activation of GLP-1 secretion is mediated *via* the PLC/IP_3_/Ca^2+^ signaling pathway, and that Gα_q_ activation in cell membranes is upstream of the PLC signaling and GLP-1 secretion elicited by EA. Cell membrane-associated G-proteins are typically activated by GPCRs. Previous studies demonstrated that the secretion of GLP-1 is largely regulated *via* GPCRs (62), which signal primarily through Gα_q_, leading to activation of PLC/Ca^2+^ signaling. Therefore, it is tempting to speculate that EA-triggered signaling in L-cells may be transmitted *via* a Gα_q_-coupled receptor. In that regard, it is possible that EA binds to the short chain fatty acid (SCFA) receptors, which are coupled to Gα_q_ to mediate SCFA-induced GLP-1 secretion from L-cells (32, 63). It was suggested that the binding of SCFAs to their receptors depends on the carboxylic acid group (64), and EA is also a carboxylic acid. In this study, we did not explore the mechanism by which EA stimulates PYY secretion. It was shown that activation Gα_q_/PLC/Ca^2+^ signaling in L-cells elicited both GLP-1 and PYY secretion (63), suggesting that EA stimulation of PYY secretion could also be mediated by this signaling pathway. However, our data show that EA at 1 μM stimulated GLP-1 secretion, whereas it was inactive at this dose in inducing PYY secretion. It is presently unclear whether this discrepancy is due to different L-cell lines used for GLP-1 (GLUTag mouse L-cells) and PYY (human NCI-H716 L-cells) secretion assays or different mechanisms involved, which remain to be determined.

In conclusion, we found for the first time that EA is a novel agent with potent anti-diabetic and anti-obesity effects in mouse models of obesity and T2D, which were associated with increased secretion of GLP-1 and PYY. These metabolically important hormones may act additively, resulting in more effective metabolic control. Mechanistically, EA directly induces incretin hormone secretion from intestinal L-cells *via* a Gα_q_/PLC-mediated mechanism (Supplementary Figure 2). Further studies are still needed to uncover the exact mechanism by which EA regulates food intake, which may play a significant role in its blood glucose lowering and anti-obesity effects. The outcomes from this research could lay the foundation for developing approaches using this small molecule for safe and effective treatment of both obesity and T2D.

## Data availability statement

The original contributions presented in the study are included in the article/Supplementary material, further inquiries can be directed to the corresponding authors.

## Ethics statement

The animal study was reviewed and approved by Institutional Animal Care and use Committee at Virginia Tech.

## Author contributions

DL and YW designed the experiments. APW, BX, RH, SZ, HM, YZ, and EG provided essential research tools. YJW, YW, AHW, and HA performed the experiments. YW and DL analyzed the data and wrote the manuscript. All authors have approved the final manuscript.
