# Supplementary figures and images for: An olive-derived elenolic acid stimulates hormone release from L-cells and exerts potent beneficial metabolic effects in obese diabetic mice
Source: Front Nutr. 2022 Nov 1;9:1051452. doi: 10.3389/fnut.2022.1051452 (PMC9664001; doi:10.3389/fnut.2022.1051452)

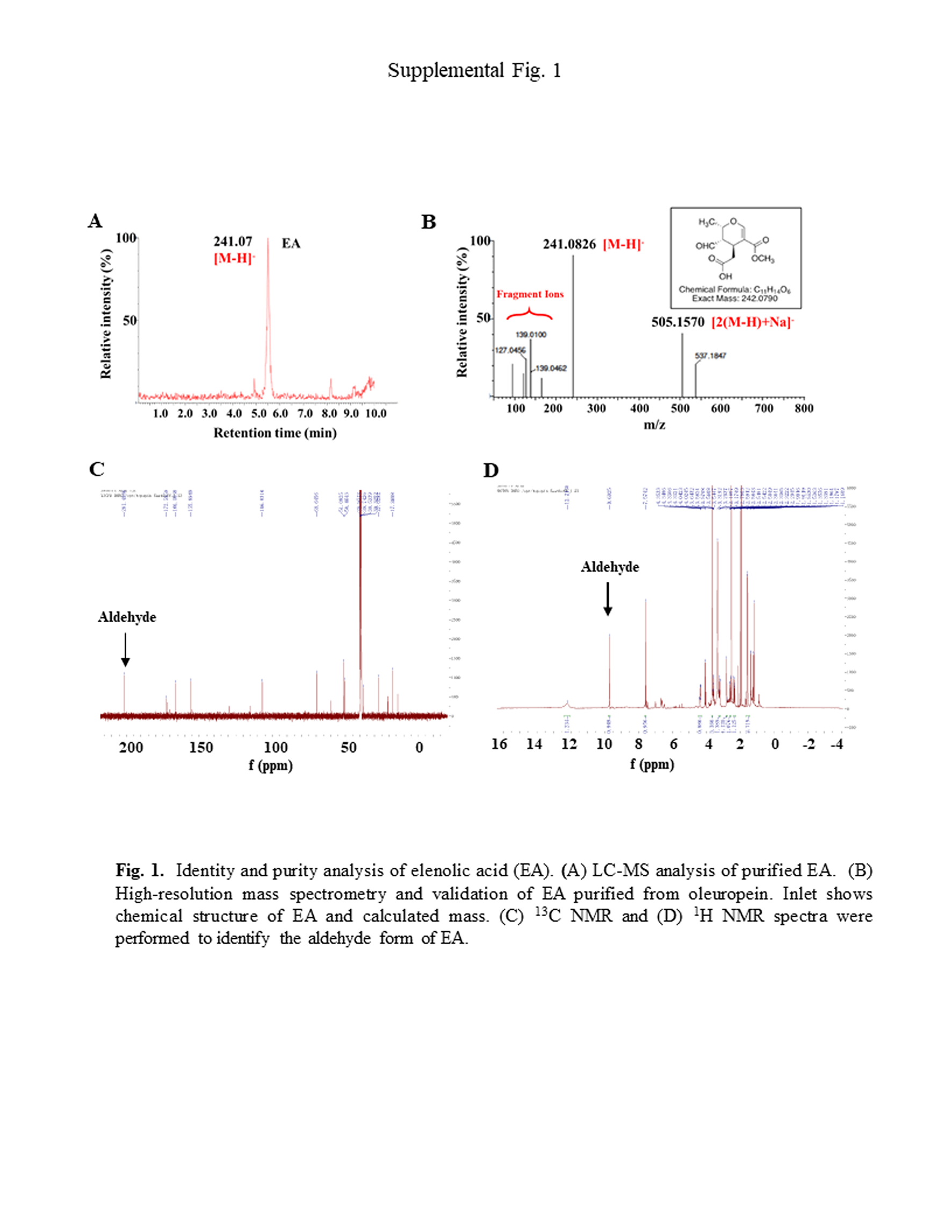

Supplement: Supplementary file 1 [file Image_1.TIF]

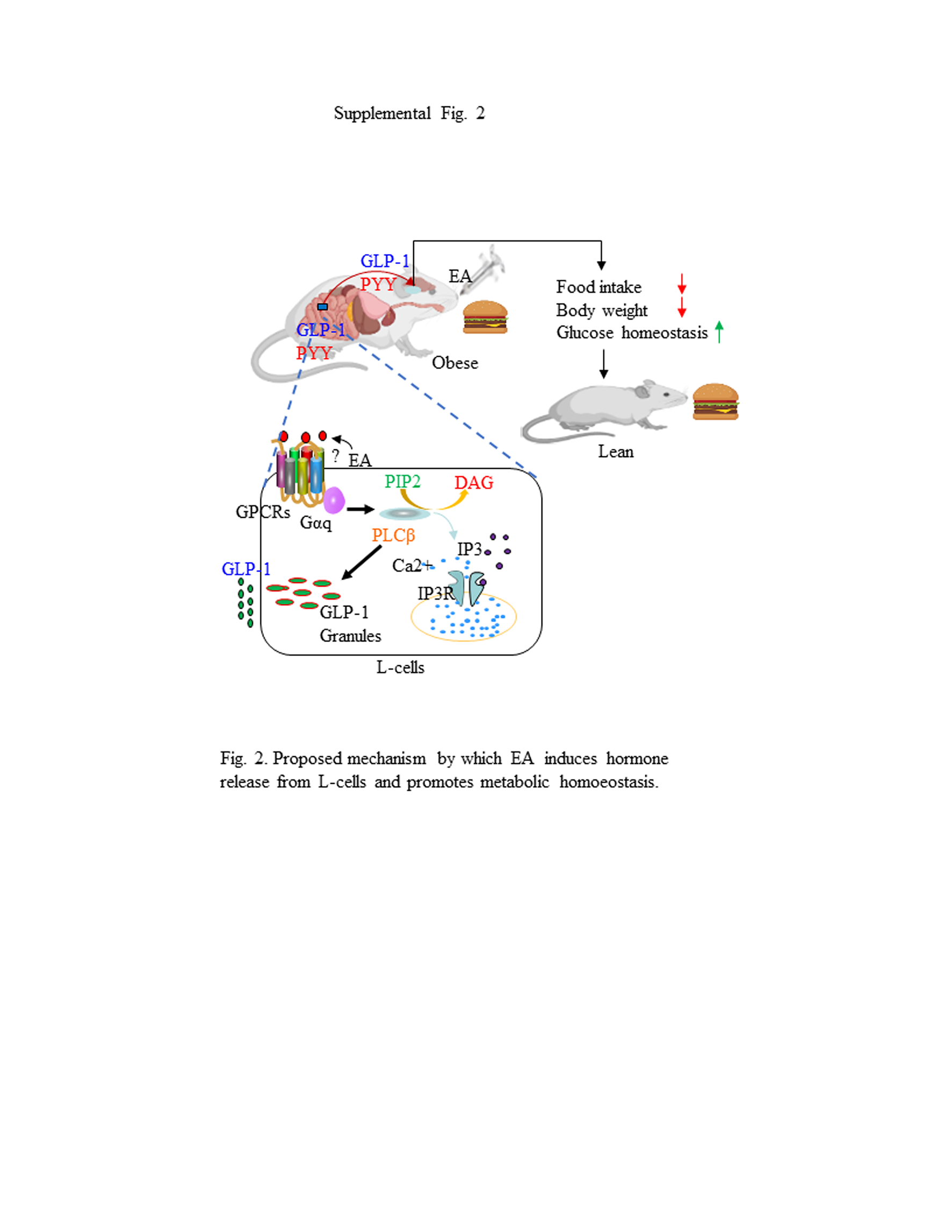

Supplement: Supplementary file 2 [file Image_2.TIF]
